# Circulating long noncoding RNA act as potential novel biomarkers for diagnosis and prognosis of non‐small cell lung cancer

**DOI:** 10.1002/1878-0261.12188

**Published:** 2018-03-25

**Authors:** Yujiao Xie, Yi Zhang, Lutao Du, Xiumei Jiang, Suzhen Yan, Weili Duan, Juan Li, Yao Zhan, Lili Wang, Shujun Zhang, Shuhai Li, Lishui Wang, Shuo Xu, Chuanxin Wang

**Affiliations:** ^1^ Department of Clinical Laboratory The Second Hospital of Shandong University Jinan China; ^2^ Department of Respiratory and Critical Care Medicine Qilu Hospital of Shandong University Jinan China; ^3^ Department of Clinical Laboratory Qilu Hospital of Shandong University Jinan China; ^4^ Department of Thoracic Surgery Qilu Hospital of Shandong University Jinan China; ^5^ Department of Neurological Surgery Qilu Hospital of Shandong University Jinan China

**Keywords:** diagnosis, long noncoding RNA, non‐small cell lung cancer, prognosis, serum, tumor biomarker

## Abstract

Lung cancer is the first leading cause of cancer deaths worldwide. Non‐small cell lung cancer (NSCLC) is the most common type of lung cancer. Increasing evidence shows that long noncoding RNA (lncRNA) are capable of modulating tumor initiation, proliferation and metastasis. In the present study, we aimed to evaluate whether circulating lncRNA could be used as biomarkers for diagnosis and prognosis of NSCLC. Expression profiles of 14 lncRNA selected from other studies were validated in 20 pairs of tissues by quantitative real‐time PCR, and the dysregulated lncRNA thus identified were further validated in serum samples from two independent cohorts along with three tumor makers (CEA, CYFRA21‐1, and SCCA). Receiver‐operating characteristic analysis was utilized to estimate the diagnostic efficiency of the candidate lncRNA and tumor markers. Importantly, we observed an association between lncRNA expression and overall survival (OS) rate of NSCLC. The expressions of SOX2 overlapping transcript (SOX2OT) and ANRIL were obviously upregulated in NSCLC tissues and serum samples compared with normal controls (*P *<* *0.01). Based on the data from the training set, we next used a logistic regression model to construct an NSCLC diagnostic panel consisting of two lncRNA and three tumor markers. The area under the curve of this panel was 0.853 (95% confidence interval = 0.804–0.894, sensitivity = 77.1%, specificity = 79.2%), and this was distinctly superior to any biomarker alone (all at *P *<* *0.05). Similar results were observed in the validation set. Intriguingly, Kaplan–Meier analysis demonstrated that low expressions of SOX2OT and ANRIL were both associated with higher OS rate (*P *=* *0.008 and 0.017, respectively), and SOX2OT could be used as an independent prognostic factor (*P *=* *0.036). Taken together, our study demonstrated that the newly developed diagnostic panel consisting of SOX2OT, ANRIL, CEA, CYFRA21‐1, and SCCA could be valuable in NSCLC diagnosis. LncRNA SOX2OT and ANRIL might be ideal biomarkers for NSCLC prognosis.

**Abbreviations**
AUC: area under the curve
CEA: carcinoembryonic antigen
CI: confidence interval
CYFRA21‐1: cytokeratin 19 fragment
lncRNA: long noncoding RNA
NSCLC: non‐small cell lung cancer
OS: overall survival
qRT‐PCR: quantitative real‐time PCR
ROC: receiver‐operating characteristic
SCCA: squamous cell carcinoma antigen
SOX2OT: SOX2 overlapping transcript

## 1 Introduction

As one of the most common fatal tumors, lung cancer is the leading cause of cancer‐related dysthanasia around the world (Chen *et al*., 2016; Fu *et al*., 2016; Siegel *et al*., 2017). Non‐small cell lung cancer (NSCLC) is the most common subtype of lung cancer, accounting for nearly 80% of lung cancers (Travis *et al*., 2015). Although patients diagnosed at an early stage can receive favorable prognosis from surgical therapy in combination with chemotherapy, radiotherapy, or targeted drug therapy, the 5‐year survival rate for most NSCLC patients remains poor with the 5‐year survival rate of 21% (Miller *et al*., 2016). Therefore, effective means for early diagnosis are imperative to reduce death. In general, individual symptoms are not evident at the early stage, imaging technologies, such as chest X‐ray and computed tomography (CT), are not definitive, and overdiagnosis and systemic cumulative radiation injuries occur occasionally (Bach *et al*., 2012; Gilbert, 2017). Although bronchoscopy in combination with pathological examination is the gold standard to diagnose NSCLC, it is not an appropriate approach to screen all individuals with respiratory diseases owing to the invasiveness and poor compliance. Currently, some serum tumor markers, such as carcinoembryonic antigen (CEA), squamous cell carcinoma antigen (SCCA) and cytokeratin 19 fragment (CYFRA21‐1), are being employed extensively to diagnose NSCLC due to the rapid detection methods available (Chen *et al*., 2015; Zhao *et al*., 2015b). However, these serum markers exhibit very low sensitivity and specificity (I and Cho, 2015). Therefore, it is urgently necessary to develop innovative and noninvasive diagnostic biomarkers that have high sensitivity and specificity.

Long noncoding RNA (lncRNA) has been the topic of debate in the realm of neoplasms as accumulating evidence has shown that lncRNA drive tumor initiation, proliferation, and metastasis (Reis and Verjovski‐Almeida, 2012; Ricciuti *et al*., 2016; Schmitt and Chang, 2016; Sun *et al*., 2017; Yang *et al*., 2015). Numerous studies have noted the differential expression of lncRNA in NSCLC and non‐neoplastic tissues/cells (Han *et al*., 2015; Yang *et al*., 2014; Zhao *et al*., 2015b). In addition, many studies have highlighted that some lncRNA steadily subsist in human serum/plasma (Qi *et al*., 2016; Shi *et al*., 2016), facilitating subsequent investigation on the role of serum/plasma lncRNA in the diagnosis and prognosis of NSCLC. However, only few serum lncRNA, such as MALAT1, HOTAIR, and GAS5, exhibit significantly different serum expression levels between NSCLC patients and healthy controls (Li *et al*., 2017; Liang *et al*., 2016; Zhang *et al*., 2017). Moreover, technical issues, such as using single or undercounted lncRNA, an insufficient specimen size, and a lack of independent validation, are often noted in such studies.

In the present study, we validated the expressions of lncRNA in two independent serum cohorts of NSCLC patients in order to identify a panel of diagnostic lncRNA. We then assessed the diagnostic efficacy of this candidate lncRNA panel. Moreover, we evaluated the association between lncRNA and prognosis of NSCLC in order to validate the feasibility of detecting NSCLC using circulating lncRNA.

## 2 Materials and methods

### 2.1 Ethical statement

All experiments were performed in accordance with relative regulations and manners. The study was approved by the Clinical Research Ethics Committee of Qilu Hospital of Shandong University. All clinical samples were collected from the Qilu Hospital of Shandong University, and written informed consent was obtained from each participant before sample collection.

### 2.2 Study design

Our study had three progressive stages: the screening stage, the training stage, and the validation stage. In the screening stage, we selected 14 lncRNA that are reportedly dysregulated in NSCLC tissue (Feng *et al*., 2015; Han *et al*., 2015; Hou *et al*., 2014; Li *et al*., 2016; Lu *et al*., 2013; Nie *et al*., 2015, 2016; Qiu *et al*., 2014; Thai *et al*., 2013; Wang *et al*., 2015; Weber *et al*., 2013; Wu *et al*., 2015; Zhang *et al*., 2014b; Zhao *et al*., 2015a) and measured their expressions in 20 paired NSCLC and matched nontumorous tissues. Next, lncRNA with aberrant expression in the tissue samples thus identified were measured in 92 paired samples (46 NSCLCs and 46 healthy controls). The lncRNA with aberrant serum expressions were further evaluated in a large cohort (140 NSCLC patients and 120 healthy controls) during the training stage. Data thus collected were combined with the currently available lung tumor markers (CEA, CYFRA21‐1, and SCCA) to construct a diagnostic panel based on a logistic regression model. The validity and diagnostic efficiency of the panel were assessed using receiver‐operating characteristic (ROC) analysis. In the validation stage, the diagnostic lncRNA panel built in the training stage was verified in another independent cohort of 100 NSCLC patients and 100 healthy controls. A Kaplan–Meier survival analysis was also conducted at the validation stage to evaluate the correlation between certain lncRNA and the overall survival (OS) rate of NSCLC patients in order to assess the prognostic power of the identified lncRNA.

### 2.3 Patients and specimens

All participants were enrolled from the Qilu Hospital of Shandong University between January 2010 and February 2012. All enrolled patients met the following inclusion criteria: Primary NSCLC was confirmed through histological examination of available samples, and no chemotherapy or radiotherapy was received before tissue/serum collection. Table S1 summarizes the clinicopathological characteristics of the patients enrolled in the training and validation stages, including sex, age, tumor size, lymph node metastasis, and TNM stage. Control samples were collected from 220 healthy subjects without any malignant tumor as indicated by normal levels of tumor markers. The demographic information of the controls is also available in Table S1. The tumors were staged according to the 8th Union of International Control of Cancer (UICC) classification (Kay *et al*., 2017).

Following surgical tumor resection, patients included in the validation set were followed up every 3 months for the first 2 years and were thereafter followed up every 6 months until March 31, 2017. Five patients were excluded during this period due to imperfect follow‐ups, and the median follow‐up time was 46 (range, 5–72) months.

Peripheral blood samples were collected from all participants before surgical resection and pharmacological intervention. Serum isolated from whole blood was immediately transferred to a 1.5‐mL Eppendorf tube and subjected to a two‐step centrifugation (1500 ***g*** for 10 min at 4 °C, and then 13 800 ***g*** for 15 min at 4 °C) to eliminate cell sediments. One aliquot of the serum sample was used within 2 h of collection to assess the expressions of tumor markers (CEA, CYFRA21‐1, and SCCA), while a second aliquot was stored at −80 °C in an RNase‐free tube until total RNA extraction.

### 2.4 Detection of common tumor markers

Serum CEA and CYFRA 21‐1 levels were detected using Elecsys and Cobas e analyzers (Roche Diagnostics, Mannheim, Germany) on Roche Cobas 6000 analyzer. Serum SCCA level was measured by ARCHITECT SCC Reagent Kit (Abbott Laboratories, Niigata, Japan) using an Abbott I2000 analyzer.

### 2.5 RNA isolation and quantitative real‐time PCR (qRT‐PCR)

Total RNA was extracted from frozen tissue by TRIzol (Invitrogen, Carlsbad, CA, USA), while RNA extraction from serum samples was accomplished using the nucleic acid purification reagent from the diagnostic kit for quantification of hepatitis C virus RNA (QIAGEN, Shenzhen, China) according to the manufacturer's instructions. RNA concentration was measured by NanoDrop spectrophotometer. The purified RNA was reversely transcribed into cDNA using PrimeScript™ RT Reagent Kit (Takara, Dalian, Liaoning, China) on a SimpliAmp™ Thermal Cycler (ABI, Singapore, Singapore). Briefly, cDNA synthesis was carried out in a 20‐μL reaction system consisting of 1 μg template RNA, 4 μL 5× PrimeScript buffer mix, 1 μL PrimeScript RT enzyme mix I, 1 μL oligo dT primer and RNase‐free dH_2_O. The reaction mixture was sequentially incubated at 37 °C for 30 min, at 85 °C for 5 s, and then at 4 °C for 60 min. qRT‐PCR was performed in a 25‐μL reaction system containing 2 μL diluted cDNA, 12.5 μL SYBR Premix Ex Taq (Takara), 0.5 μL DyeII, 0.75 μL each of the forward and reverse primers, and 8.5 μL nuclease‐free water. Amplifications were conducted on a CFX96™ Real‐Time System (Bio‐Rad Laboratories, Hercules, CA, USA). Briefly, after an initial denaturation step at 95 °C for 30 s, 42 amplification cycles were carried out with a melting temperature of 95 °C for 5 s and an annealing temperature of 58 °C for 30 s. This was followed by a melting curve analysis to ensure the specificity of the PCR products. Each experiment was performed in triplicate. GAPDH was selected as the housekeeping gene, and the relative expressions of lncRNA were calculated using the $2^{-\Delta \Delta C_{t}}$ method. The amplicons of target genes were identified after gel extraction and sequencing using PubMed gene blast.

### 2.6 Statistical analysis

All lncRNA expression data were assessed by spss statistics 22.0 (IBM, Chicago, IL, USA) to ascertain normal distribution. Nonparametric Mann–Whitney *U*‐test was used to compare the differences in lncRNA expression between the tumor and control groups. Scatter diagrams were generated with graphpad prism 5 (San Diego, CA, USA). Logistic regression analysis was performed utilizing matlab software (MATLAB, R2014a, Natick, MA, USA) to establish the lncRNA panel. ROC curves were generated using medcalc 15.2.2 (MedCalc, Mariakerke, Belgium), and the area under the curve (AUC) was utilized to evaluate the feasibility of using the lncRNA to detect NSCLC. Survival curves were generated using the Kaplan–Meier method, and survival differences were estimated by a log‐rank test. Multivariate Cox regression analysis was performed to identify significantly independent prognostic factors. A *P*‐value < 0.05 was considered statistically significant.

## 3 Results

### 3.1 Discovery and validation of dysregulated lncRNA in tissue and serum

Based on previous studies, we selected 14 aberrant NSCLC‐related lncRNA as potential diagnostic candidates in the present study. A total of 20 paired NSCLC and matched adjacent normal tissues were used to validate the relative expressions of these 14 NSCLC‐related lncRNA by qRT‐PCR. Our data showed that 12 lncRNA were significantly dysregulated in NSCLC tissues compared with adjacent normal tissues (Table S2). Next, we assessed the expressions of the 12 lncRNA in 92 serum samples (46 NSCLCs and 46 healthy controls) and identified two lncRNA [SOX2 overlapping transcript (SOX2OT) and ANRIL] that were overexpressed in tumor serum samples compared with healthy controls (Table S3, all at *P *<* *0.01).

### 3.2 Verification of expression of the selected lncRNA in serum

**Figure 1:**
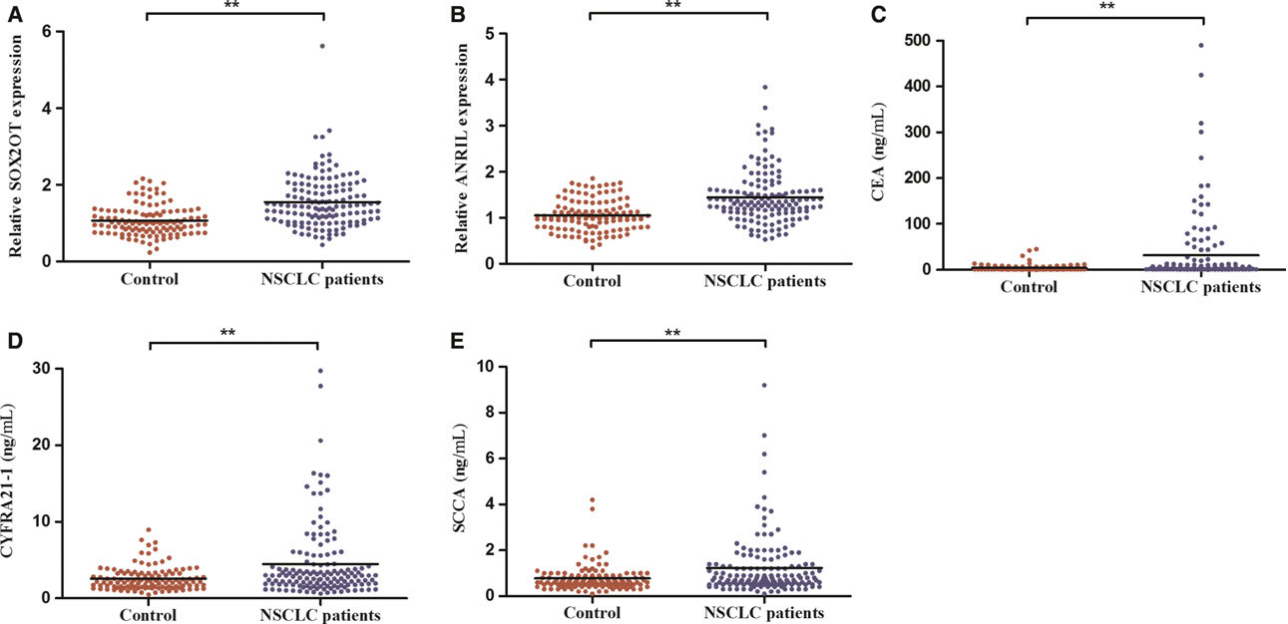
Expression levels of SOX2OT, ANRIL, CEA, CYFRA21‐1, and SCCA in serum samples of NSCLC patients and healthy controls during the training stage. SOX2OT and ANRIL were upregulated in the serum of NSCLC patients compared to healthy controls, as tested by qRT‐PCR (A, B). CEA, CYFRA21‐1, and SCCA were overexpressed in NSCLC patients compared with healthy controls (C–E). **P <* *0.01.

**Figure 2:**
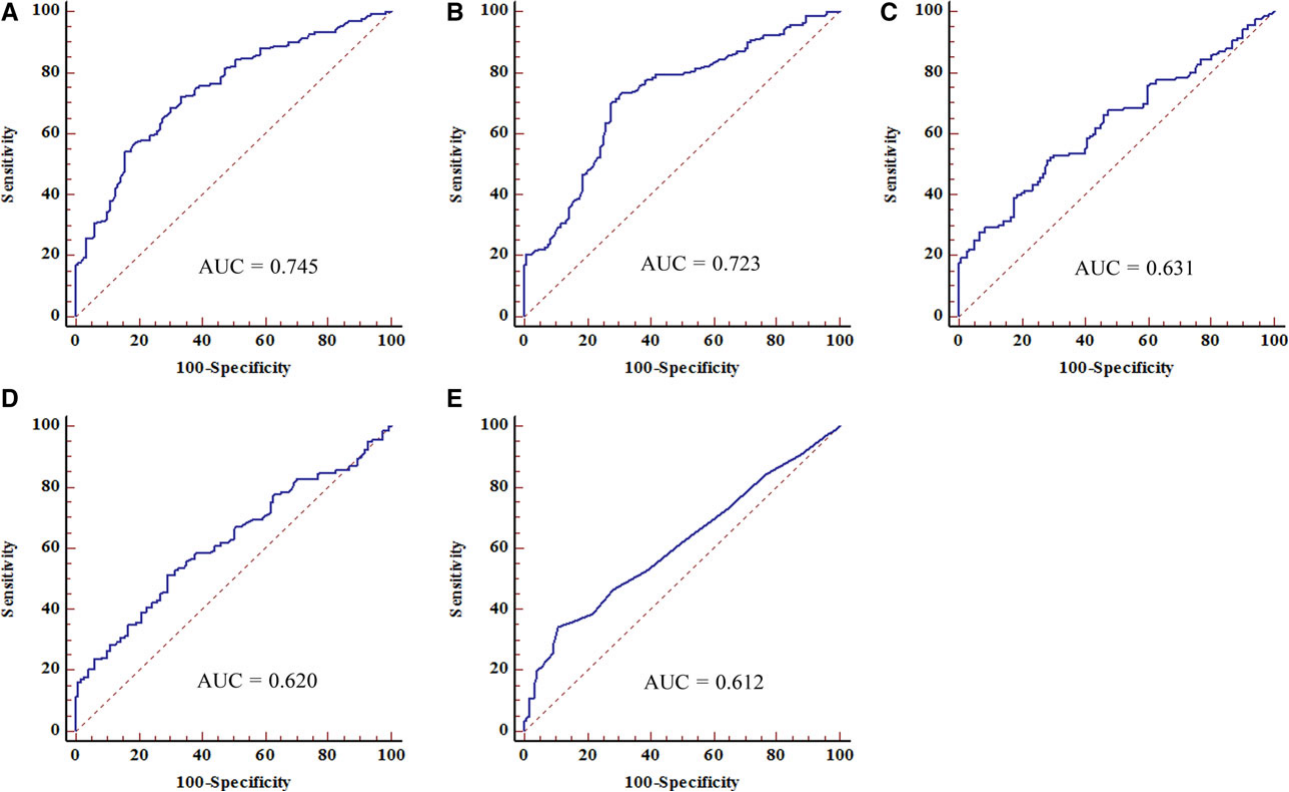
ROC analyses of SOX2OT, ANRIL, CEA, CYFRA21‐1, and SCCA during the training stage for diagnosis of NSCLC. The AUCs of SOX2OT (A) and ANRIL (B) were greater than those of CEA (C), CYFRA21‐1 (D), and SCCA (E), *P *<* *0.05.

We then increased the sample size to include 260 serum specimens (140 NSCLC patients and 120 healthy controls) in the training set to verify the expressions of SOX2OT and ANRIL by qRT‐PCR. We confirmed the overexpression of SOX2OT and ANRIL in the serum of NSCLC patients compared with controls (Fig. 1A,B). Concurrently, we evaluated the expression levels of three tumor markers (CEA, CYFRA21‐1, and SCCA) in the training set (Fig. 1C–E). ROC was used to evaluate the diagnostic efficacy of the two lncRNA and the three tumor markers currently employed for NSCLC detection. The AUCs of SOX2OT, ANRIL, CEA, CYFRA21‐1, and SCCA were 0.745 [95% confidence interval (CI) = 0.688–0.797], 0.723 (95% CI = 0.665–0.777), 0.631 (95% CI = 0.570–0.690), 0.620 (95% CI = 0.558–0.680), and 0.612 (95% CI = 0.550–0.672), respectively (Fig. 2).

### 3.3 Construction of a diagnostic lncRNA panel for NSCLC

**Figure 3:**
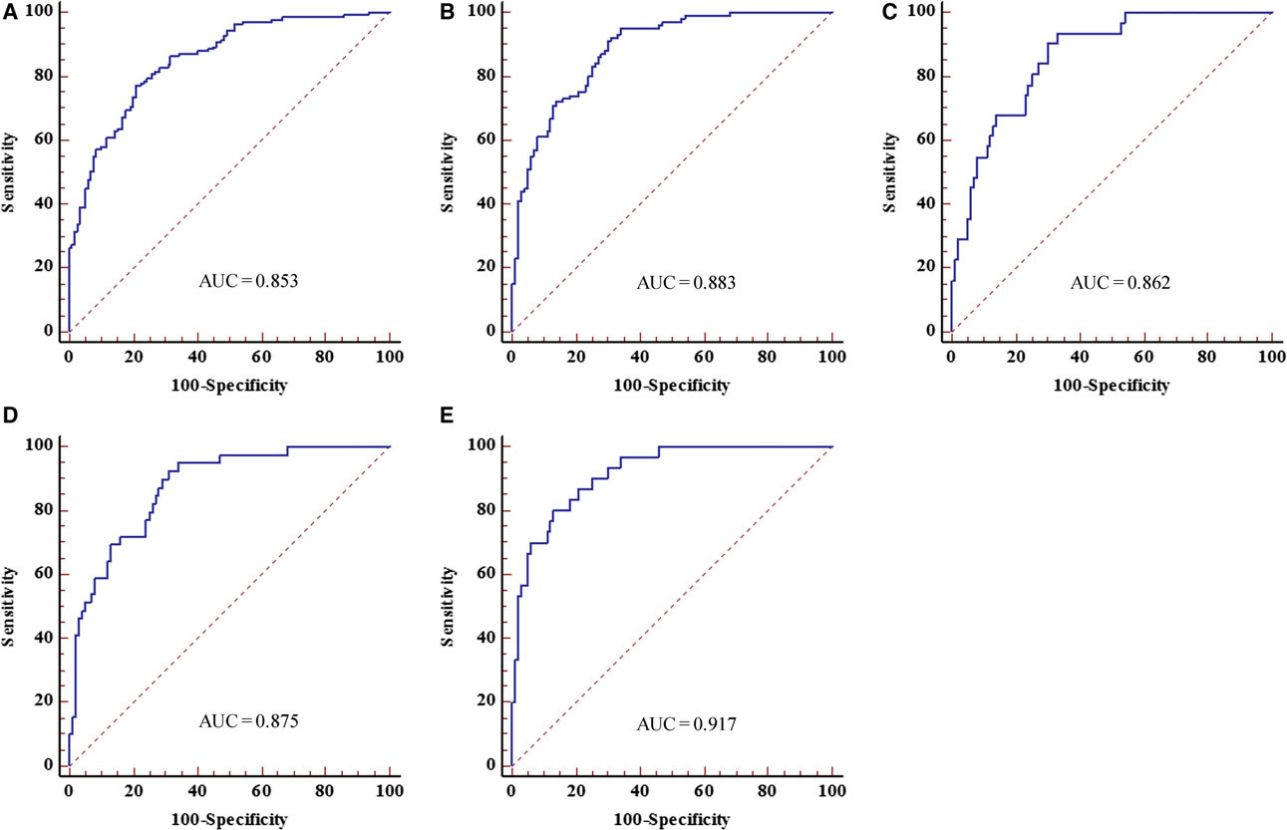
ROC analyses of the diagnostic panel consisting of SOX2OT, ANRIL, CEA, CYFRA21‐1, and SCCA for the diagnosis of NSCLC. The AUCs of the diagnostic panel for the diagnosis of NSCLC in the training set (A) and validation set (B) were calculated by ROC analysis. The AUCs of the diagnostic panel for patients with TNM stage I (C), II (D), and III (E) in validation set were performed by ROC analysis.

Based on the training set, a logistic regression model was established to diagnose NSCLC. The probability of predicting NSCLC from the 5‐molecule panel was enumerated using following formula: Logit (*P*) = 1.6487 − (0.002 × CEA) − (0.0249 × CYFRA21‐1) − (0.095 × SCCA) − (0.5474 × SOX2OT) −  (0.6174 × ANRIL). We constructed the diagnostic 5‐molecule panel from the training set using this formula and obtained an AUC of 0.853 (95% CI = 0.804–0.894, sensitivity = 77.1%, specificity = 79.2%, Fig. 3A). The predictive capability of the panel was significantly higher compared with that of any molecule alone (all at *P *<* *0.05).

### 3.4 Validation of the constructed lncRNA panel

We further validated the expression pattern of SOX2OT and ANRIL lncRNA and the efficiency of the 5‐molecule panel estimated during the training stage by employing another independent validation set (100 NSCLC patients and 100 controls). No significant differences were observed in the distribution of age, sex, and tumor characteristics for the NSCLC and control samples between the training and validation sets (Table S1). Similarly, SOX2OT and ANRIL were clearly upregulated in the validation set (Fig. S1A,B). The expressions of CEA, CYFRA21‐1, and SCCA in the validation set are shown in Fig. S1C–E.

The AUCs of SOX2OT, ANRIL, CEA, CYFRA21‐1, and SCCA in the validation set were 0.740 (95% CI = 0.673–0.799), 0.723 (95% CI = 0.656–0.784), 0.622 (95% CI = 0.551–0.689), 0.622 (95% CI = 0.551–0.690), and 0.607 (95% CI = 0.536–0.675), respectively (Fig. S2). The AUC of the 5‐molecule panel in the validation set was 0.883 (95% CI = 0.830–0.924, sensitivity = 91.0%, specificity = 70.0%), which was statistically higher than that of any single molecule (all at *P *<* *0.05) (Fig. 3B). In addition, we calculated the diagnostic performance of this panel in distinguishing NSCLC patients with different TNM stages from healthy individuals in the validation set. The AUCs of the panel for patients with TNM stages I, II, and III were 0.862 (95% CI = 0.790–0.916), 0.875 (95% CI = 0.808–0.925), and 0.917 (95% CI = 0.855–0.958), respectively (Fig. 3C–E).

### 3.5 Stability of SOX2OT and ANRIL in serum

The serum from NSCLC patients was subjected to harsh conditions to evaluate the stability of SOX2OT and ANRIL. To this end, five serum samples were incubated at room temperature for various durations (0, 3, 6, 12, and 24 h) or were subjected to repetitive freeze–thaw cycles (1, 4, 6, 8 and 10). RNA was then isolated, and qRT‐PCR was performed to test the expressions of SOX2OT and ANRIL. The data showed imperceptible changes in the relative expressions of SOX2OT and ANRIL under the two conditions tested (Fig. S3).

### 3.6 Correlation between the two serum lncRNA and clinicopathological characteristics

Table 1 lists the correlation between SOX2OT and ANRIL expression levels in serum and the clinicopathological characteristics of the NSCLC patients in the validation set. Higher levels of serum SOX2OT were remarkably correlated with lymph node metastasis (*P *<* *0.05). However, we did not observe any association between the expressions of the lncRNA and patient age, sex, tumor size, or TNM stage.

**Table 1:** Correlation between serum lncRNA concentrations and clinicopathological characteristics of patients with NSCLC in validation set [median (interquartile range)]

Parameters	Total cases	SOX2OT	*P* value	ANRIL	*P* value
Age (years)					
≤ 61	57	1.27 (1.01–1.50)	0.05	1.38 (1.04–1.87)	0.71
> 61	43	1.34 (1.19–1.74)		1.32 (1.04–1.72)	
Sex					
Male	65	1.33 (1.17–1.63)	0.15	1.32 (1.05–1.75)	0.90
Female	35	1.18 (0.95–1.62)		1.36 (1.01–1.83)	
Tumor size					
≤ 3 cm	44	1.27 (1.01–1.62)	0.54	1.24 (0.97–1.72)	0.17
> 3 cm	56	1.31 (1.16–1.62)		1.43 (1.10–1.85)	
Lymph node metastasis					
Negative	52	1.22 (0.97–1.52)	0.01	1.21 (0.97–1.72)	0.08
Positive	48	1.40 (1.22–1.70)		1.41 (1.12–1.87)	
TNM stage					
I	31	1.22 (0.96–1.58)	0.33	1.18 (0.96–1.72)	0.30
II	39	1.28 (1.05–1.53)		1.25 (1.04–1.69)	
III	30	1.38 (1.22–1.64)		1.58 (1.14–1.91)	

### 3.7 Association between serum lncRNA and OS rate

**Figure 4:**
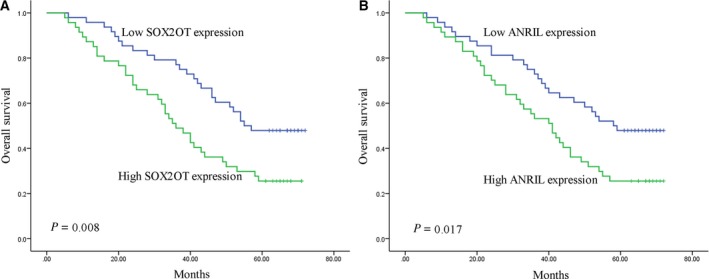
Survival analyses of NSCLC patients stratified by median lncRNA expression level. Kaplan–Meier curves show that patients with low SOX2OT (A) or low ANRIL (B) expression had higher survival rate in the validation set.

A total of 100 NSCLC patients (including five patients who were excluded during the follow‐up period) were followed up for a mean duration of 46 (range 5–72) months. Of the 95 NSCLC patients, 60 (63.2%) died within 5 years of surgery, and the 5‑year OS rate was 36.8%. Kaplan–Meier survival analysis indicated that patients with low expressions of SOX2OT (*P* = 0.008, Fig. 4A) and ANRIL (*P* = 0.017, Fig. 4B) achieved a higher 5‐year OS rate. Univariate Cox proportional hazards regression model analysis revealed a statistically significant correlation between the OS rate of NSCLC patients and SOX2OT level (*P* = 0.009), ANRIL level (*P = *0.019), tumor size (*P < *0.001), lymph node metastasis (*P < *0.001), and TNM stage (*P < *0.001) (Table 2). Above‐mentioned parameters were further analyzed using a multivariate analysis, and SOX2OT level (*P = *0.036) and TNM stage (*P = *0.023) were identified as statistically significant prognostic factors (Table 2).

**Table 2:** Univariate and multivariate analyses for OS prediction in validation cohort. HR, hazard ratio

Parameters	Categories	Univariate analysis		Multivariate analysis	
		HR (95% CI)	*P* value	HR (95% CI)	*P* value
Age (years)	≤ 61, > 61	1.335 (0.803–2.220)	0.265		
Sex	Male, female	0.586 (0.330–1.040)	0.068		
Tumor size	≤ 3 cm, > 3 cm	3.071 (1.720–5.481)	< 0.001	1.375 (0.725–2.607)	0.329
Lymph node metastasis	Negative, positive	4.530 (2.570–7.983)	< 0.001	1.638 (0.745–3.601)	0.220
TNM stage	I, II, III	2.788 (1.961–3.966)	< 0.001	1.859 (1.089–3.175)	0.023
SOX2OT expression	Low, high	1.988 (1.187–3.330)	0.009	1.793 (1.040–3.093)	0.036
ANRIL expression	Low, high	1.856 (1.108–3.109)	0.019	1.507 (0.874–2.598)	0.140

## 4 Discussion

In this study, we initially selected 14 lncRNA from previously published NSCLC‐related studies and then validated their dysregulated expressions in NSCLC tissue samples. Among 14 lncRNA tested, we confirmed the dysregulation of 12 lncRNA in NSCLC tissues, corroborating that our results were consistent with previous studies. We next assessed the expression levels of the 12 aberrant lncRNA in serum and observed that two lncRNA (SOX2OT and ANRIL) were significantly upregulated in NSCLC patients compared with healthy controls. Intriguingly, the AUCs of SOX2OT and ANRIL were both greater than those of the currently used tumor markers (CEA, CYFRA21‐1, and SCCA), indicating remarkable efficacy of SOX2OT and ANRIL in distinguishing NSCLC patients from controls. Based on this result, we constructed a diagnostic panel consisting of the two lncRNA and the three tumor markers, which could distinguish NSCLC patients from healthy controls with significantly higher AUC. In addition, circulating SOX2OT was identified as an independent factor for NSCLC prognosis.

To the best of our knowledge, we are the first to report the expression profile of serum lncRNA in a large sample set (460 specimens) with two independent sets. A previous study by Hu *et al*. (2016) has reported the expression profile of lncRNA in NSCLC plasma; however, their sample size was relatively small (140 specimens). Moreover, most of the previous studies of NSCLC‐related lncRNA focus on single molecules when exploring the potential of lncRNA as novel tumor biomarkers. However, multiple factors may jointly portray their roles in tumor initiation, development, and metastasis progress. Importantly, one lncRNA is too inadequate to act as a novel malignant tumor biomarker; nevertheless, the commonly used tumor markers have lower sensitivity in diagnosing NSCLC. In our study, lncRNA (SOX2OT and ANRIL) and tumor markers (CEA, CYFRA21‐1, and SCCA) were combined into a single diagnostic panel after a logistic regression analysis in the training and validation sets. Our subsequent analysis showed that in the same cohort, this diagnostic panel possessed a superior diagnostic capacity compared with the use of the five molecules separately. Therefore, this panel‐based, integrated analysis provided a multidimensional interpretation to recognize new cases of NSCLC. Besides, our results clearly depicted the excellent sensitivity and specificity of this panel in diagnosing NSCLC, especially at an early stage based on the AUC of the panel in NSCLC patients at different TNM stages.

In this study, we demonstrated a significant association between SOX2OT expression and lymph node metastasis which is a vital prognostic feature for NSCLC patients. Furthermore, NSCLC patients with low expressions of SOX2OT and ANRIL had worse OS rate than those with high expression levels. To exclude the mutual impact of the various factors associated with prognosis, multivariate survival analysis revealed that only TNM stage and SOX2OT could act as independent prognostic factors for NSCLC. Interestingly, SOX2OT and ANRIL expressions have previously been linked with cancers by others. Hou *et al*. (2014) have reported SOX2OT to be a prognostic indicator of poor survival in lung cancer. ANRIL has been recently identified as an independent prognostic factor for disease‐free survival and OS in patients with gastric cancer (Zhang *et al*., 2014a). SOX2OT and ANRIL are considered as potential prognosis biomarkers in these studies.

This study utilized serum which is easily obtainable. Some studies have hypothesized that lncRNA can be released into various body fluids, including serum, saliva, and urine, as a result of cancer cell excretion. We next performed assays to assess the stability of the serum lncRNA. Our results indicated that the expressions of lncRNA did not change significantly even when serum samples were exposed to harsh conditions. Researchers have proposed at least three mechanisms that can explain the stability of lncRNA in serum: (a) lncRNA may be selectively enclosed into small, membrane‐covered vesicles, such as exosomes, microparticles, and apoptotic bodies; (b) lncRNA may fold into complex secondary and tertiary structures; or (c) lncRNA may bind with protein to avoid degradation (Hewson and Morris, 2016; Shi *et al*., 2016). Our results and these possible stabilizing mechanisms further underscored that serum SOX2OT and ANRIL may serve as potential novel biomarkers for diagnosis and prognosis of NSCLC.

Previous functional studies on lncRNA provide strong evidence that SOX2OT and ANRIL are potential biomarkers. SOX2OT is involved in various stages of progression of a malignant tumor. Splice variants (SOX2OT‐S1 and SOX2OT‐S2) have been identified to co‐upregulate with SOX2 and OCT4 (two key regulators of pluripotency) in esophageal squamous cell carcinoma (Shahryari *et al*., 2014). SOX2OT has also been found to be concomitantly overexpressed along with SOX2 and OCT4A in lung tumor samples (Saghaeian Jazi *et al*., 2016). SOX2OT may also be involved in cell differentiation and may thus influence tumorigenesis. Furthermore, SOX2OT is also involved in other biological characteristics of multiple pernicious tumors, including development and metastasis (Askarian‐Amiri *et al*., 2014; Shi, 2015; Zhang *et al*., 2016). Similarly, ANRIL also plays a crucial role in tumor evolution. Nie *et al*. (2015) have suggested that ANRIL controls the proliferation, apoptosis, and migration of NSCLC cells by silencing KLF2 and P21 in the EZH2 pathway. Additionally, Sun *et al*. (2016) have reported that ANRIL knockdown significantly suppresses cell migration and invasion, tumor growth, and the capacity for lymphatic metastasis, suggesting that ANRIL is a potential curative target in colorectal cancer. Qiu *et al*. (2016) have shown that ANRIL can decrease P15 expression and increase Bcl‐2 expression, thereby accelerating cell growth and suppressing apoptosis and senescence, eventually leading to the proliferation of epithelial ovarian cancer cells. These reports further strengthen our finding that circulating lncRNA SOX2OT and ANRIL have immense potential to serve as NSCLC biomarkers.

In our study, only two lncRNA (SOX2OT and ANRIL) were significantly overexpressed in NSCLC serum, which was inconsistent with other tissue/cell studies. We attributed this discrepancy to the sample type (tissue versus serum), sample size, and the demographic characteristics of the study population (ethnicity, region, etc.), as well as the testing methods employed. Most participants in our study were enrolled from the same hospital, which makes a limitation of our study. Further multicenter cohort should be carried out in future to further strengthen these results. As SOX2OT has a variety of transcript isoforms that play different roles in tumor progression, sequencing should be performed to further identify the functional isoforms. We validated the diagnostic potential of a 5‐molecule panel to distinguish NSCLC patients from healthy controls. However, we did not assess the two lncRNA in serum samples of patients with other malignant diseases or benign respiratory diseases. This will be important to explore the specificity of SOX2OT and ANRIL and warrant further investigation.

## 5 Conclusions

We established a diagnostic panel consisting of lncRNA (SOX2OT and ANRIL) and tumor markers (CEA, CYFRA21‐1, and SCCA), which could diagnose NSCLC with superior sensitivity and specificity. In addition, we identified SOX2OT as an independent predictor of NSCLC survival rate. Further studies on samples from diverse regions and ethnic groups using standardized detection methods should be conducted to confirm the usefulness of lncRNA as promising potential biomarkers for diagnosis and prognosis of NSCLC.

## Author contributions

CW designed this study. YX, YZ, LD, XJ, SY, and YZ performed all the experiments. YX, LW, JL, WD, LW, and SZ evaluated the data. CW, SX, SL, YX, and YZ wrote the manuscript. All authors read and approved the final manuscript.

## Supporting information


**Fig. S1.** Expression levels of SOX2OT, ANRIL, CEA, CYFRA21‐1, and SCCA in serum samples of NSCLC patients and healthy controls during the validation stage.

**Fig. S2.** ROC analyses of SOX2OT, ANRIL, CEA, CYFRA21‐1, and SCCA during the validation stage for NSCLC diagnosis.

**Fig. S3.** Stability of SOX2OT and ANRIL in serum.

**Table S1.** Clinicopathological characteristics of patients and demographic information of controls in training set and validation set.

**Table S2.** The selected lncRNA concentration in 20 paired NSCLC and adjacent normal tissues [median (interquartile range)].

**Table S3.** The selected lncRNA concentration in NSCLC serum of 46‐samples compared to the controls [median (interquartile range)].
