# Supplementary figures and images for: Circulating long noncoding RNA act as potential novel biomarkers for diagnosis and prognosis of non‐small cell lung cancer
Source: Mol Oncol. 2018 Mar 25;12(5):648–58. doi: 10.1002/1878-0261.12188 (PMC5928376; doi:10.1002/1878-0261.12188)

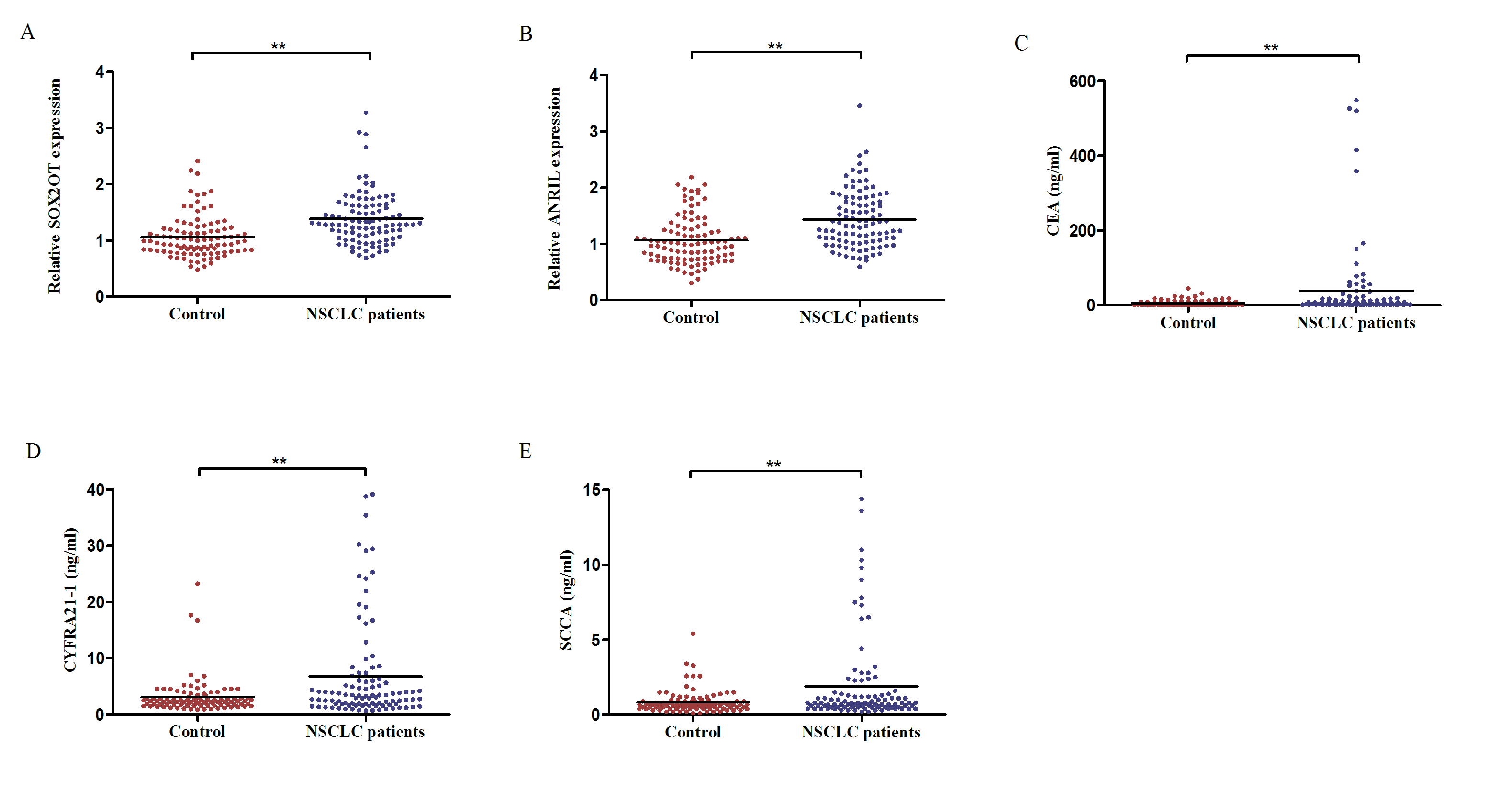

Supplement: Supplementary file 1 — Fig. S1. Expression levels of SOX2OT, ANRIL, CEA, CYFRA21‐1, and SCCA in serum samples of NSCLC patients and healthy controls during the validation stage. [file MOL2-12-648-s001.tif]

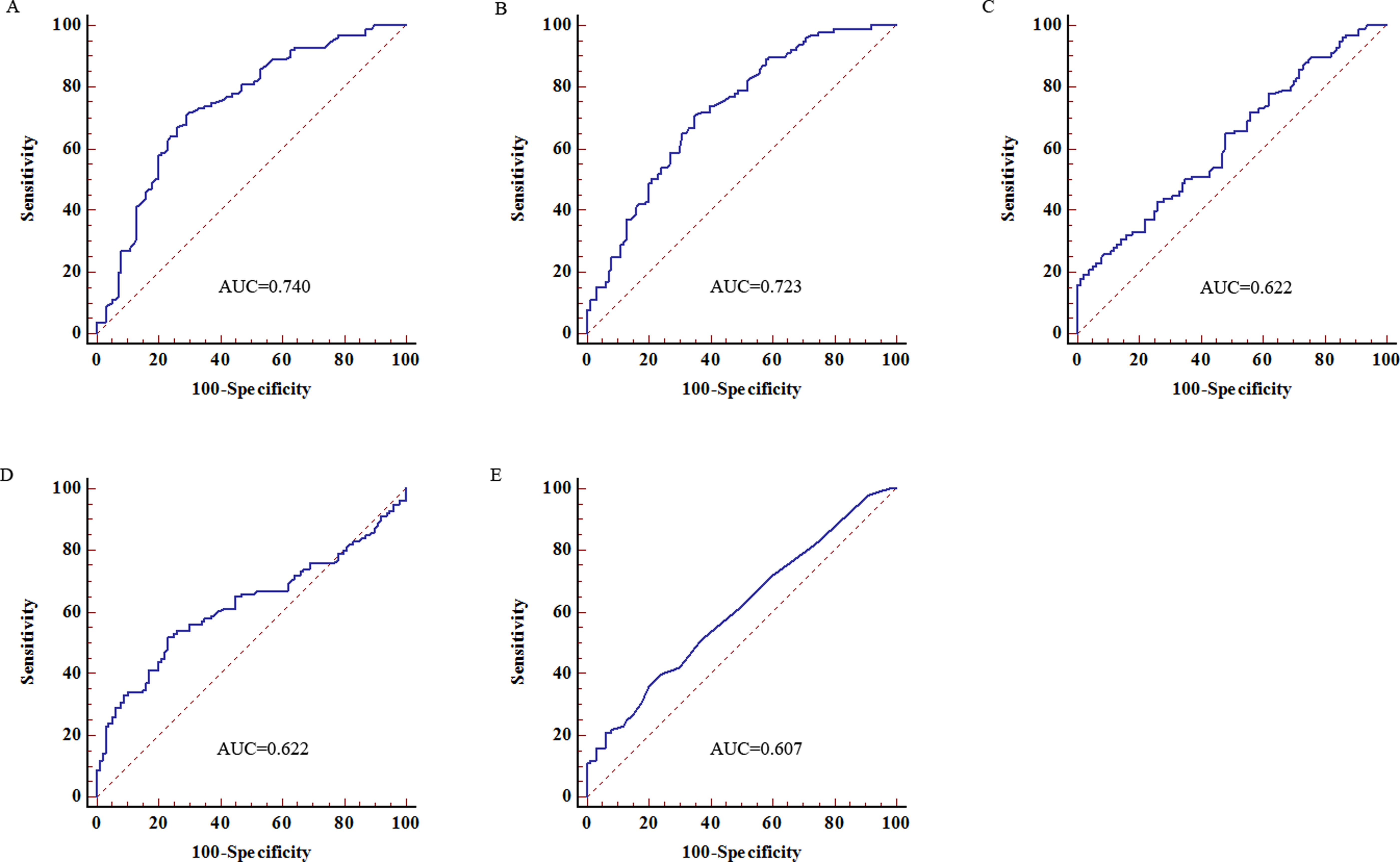

Supplement: Supplementary file 2 — Fig. S2. ROC analyses of SOX2OT, ANRIL, CEA, CYFRA21‐1, and SCCA during the validation stage for NSCLC diagnosis. [file MOL2-12-648-s002.tif]

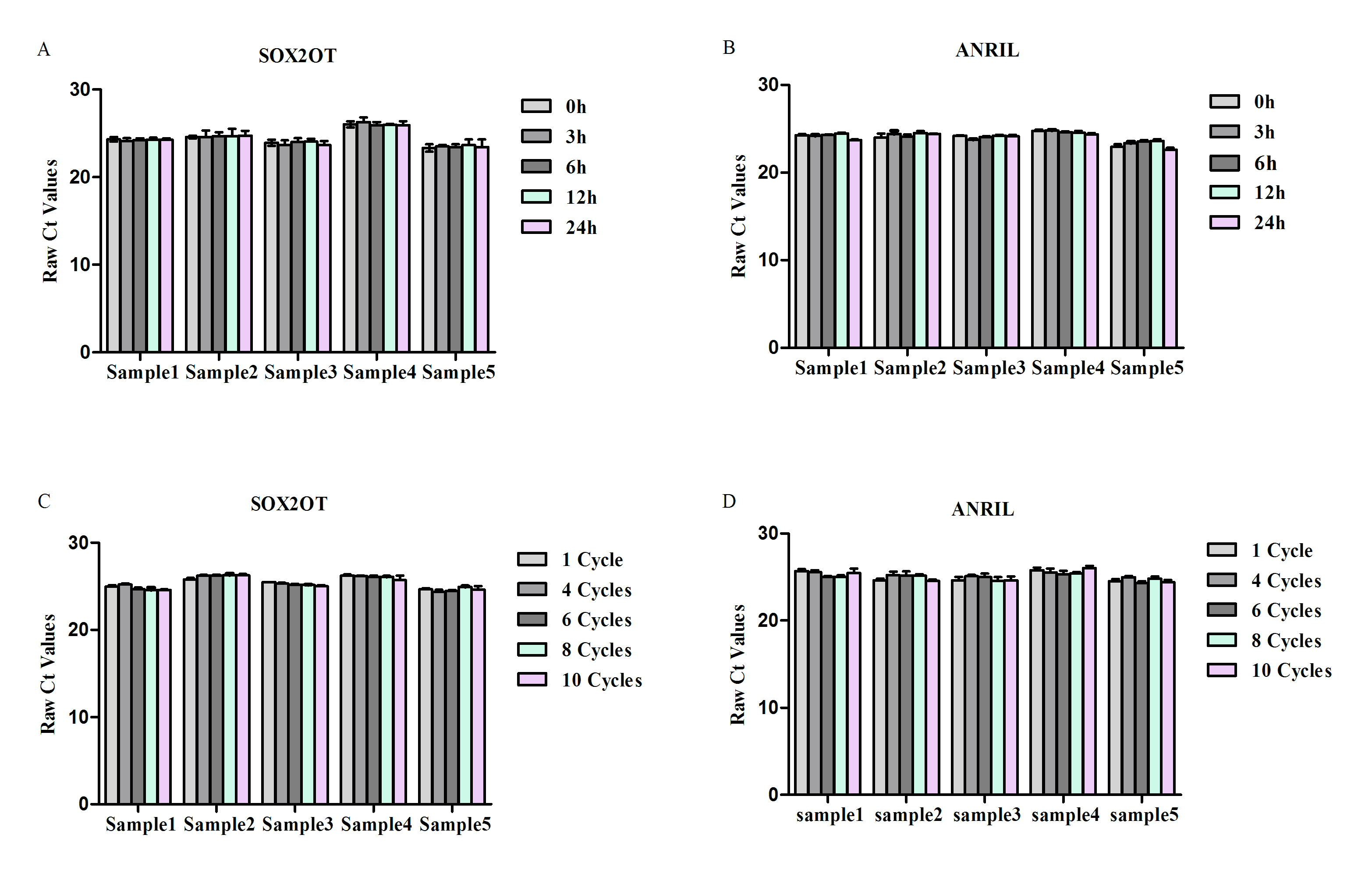

Supplement: Supplementary file 3 — Fig. S3. Stability of SOX2OT and ANRIL in serum. [file MOL2-12-648-s003.tif]
