# Supplementary material for: Circulating long noncoding RNA act as potential novel biomarkers for diagnosis and prognosis of non‐small cell lung cancer
Source: Mol Oncol. 2018 Mar 25;12(5):648–58. doi: 10.1002/1878-0261.12188 (PMC5928376; doi:10.1002/1878-0261.12188)
Supplement: Supplementary file 4 — Table S1. Clinicopathological characteristics of patients and demographic information of controls in training set and validation set. [file MOL2-12-648-s004.docx]

**Table S1. Clinicopathological characteristics of patients and demographic information of controls in training set and validation set.**

| **Variable** | **Training set** | **Validation set** | **P value** |
| --- | --- | --- | --- |
|  | **n=260** | **n=200** |  |
| Control (number) | 120 | 100 |  |
| Age(years) ^a^ |  |  | 0.08 |
| ≤53 | 61 | 39 |  |
| >53 | 59 | 61 |  |
| Sex |  |  | 0.76 |
| Male | 72 | 62 |  |
| Female | 48 | 38 |  |
| NSCLC(number) | 140 | 100 |  |
| Age(years) ^b^ |  |  | 0.39 |
| ≤61 | 72 | 57 |  |
| >61 | 68 | 43 |  |
| Sex |  |  | 0.57 |
| Male | 86 | 65 |  |
| Female | 54 | 35 |  |
| Tumor size(diameter) |  |  | 0.97 |
| ≤3cm | 62 | 44 |  |
| >3cm | 78 | 56 |  |
| Lymph node matastasis |  |  | 0.22 |
| Negative | 84 | 52 |  |
| Positive | 56 | 48 |  |
| TNM stage |  |  | 0.28 |
| Ⅰ | 57 | 31 |  |
| Ⅱ | 50 | 39 |  |
| Ⅲ | 33 | 30 |  |

NSCLC: non-small cell lung cancer. ^a^ Median of control participants in training set. ^b^ Median of patients in training set.
