# Supplementary material for: Circulating long noncoding RNA act as potential novel biomarkers for diagnosis and prognosis of non‐small cell lung cancer
Source: Mol Oncol. 2018 Mar 25;12(5):648–58. doi: 10.1002/1878-0261.12188 (PMC5928376; doi:10.1002/1878-0261.12188)
Supplement: Supplementary file 5 — Table S2. The selected lncRNA concentration in 20 paired NSCLC and adjacent normal tissues [median (interquartile range)]. [file MOL2-12-648-s005.docx]

**Table S2. The selected lncRNA concentration in 20 paired NSCLC and adjacent normal tissues [median (interquartile range)]**

| **LncRNA** | **Control** | **NSCLCs** | ***P*** | **LncRNA** | **Control** | **NSCLCs** | ***P*** |
| --- | --- | --- | --- | --- | --- | --- | --- |
| SOX2OT | 1.09(0.32-3.78) | 4.01(2.40-5.74) | 0.02 | Linc01207 | 0.94(0.59-3.84) | 4.67(3.58-5.50) | <0.01 |
| ANRIL | 0.64(0.25-3.83) | 7.18(0.96-11.59) | <0.01 | UCA1 | 0.81(0.29-5.38) | 6.74(4.35-9.11) | 0.02 |
| IRAIN | 1.20(0.61-2.68) | 4.92(1.58-26.78) | <0.01 | MALAT1 | 0.81(0.41-2.31) | 3.69(1.21-5.62) | 0.03 |
| PCAT1 | 0.45(0.10-11.46) | 9.10(0.82-13.83) | 0.03 | RGMB-AS1 | 1.70(0.19-4.90) | 5.62(3.55-10.28) | 0.01 |
| CCAT2 | 1.35(0.25-5.01) | 5.11(3.11-12.28) | 0.02 | TUG1 | 1.04(0.53-1.88) | 0.42(0.24-0.87) | 0.03 |
| SCAL1 | 1.02(0.38-4.60) | 3.69(2.20-5.44) | 0.06 | PANDAR | 1.01(0.59-2.24) | 0.57(0.19-1.15) | 0.04 |
| MEG3 | 0.64(0.23-1.58) | 0.26(0.14-0.57) | 0.08 | HNF1A-AS1 | 1.17(0.57-3.27) | 4.59(3.60-7.36) | 0.02 |
