# Supplementary material for: Circulating long noncoding RNA act as potential novel biomarkers for diagnosis and prognosis of non‐small cell lung cancer
Source: Mol Oncol. 2018 Mar 25;12(5):648–58. doi: 10.1002/1878-0261.12188 (PMC5928376; doi:10.1002/1878-0261.12188)
Supplement: Supplementary file 6 — Table S3. The selected lncRNA concentration in NSCLC serum of 46‐samples compared to the controls [median (interquartile range)]. [file MOL2-12-648-s006.docx]

**Table S3. The selected lncRNA concentration in NSCLC serum of 46-samples compared to the controls [median (interquartile range)].**

| **LncRNA** | **Control** | **NSCLCs** | ***P*** | **LncRNA** | **Control** | **NSCLCs** | ***P*** |
| --- | --- | --- | --- | --- | --- | --- | --- |
| SOX2OT | 0.88(0.72-1.05) | 1.71(1.31-2.30) | <0.01 | TUG1 | 0.99(0.65-1.54) | 1.20(0.69-1.53) | 0.61 |
| ANRIL | 0.95(0.77-1.09) | 1.26(1.00-1.55) | <0.01 | UCA1 | 1.09(0.68-1.38) | 1.14(0.80-1.62) | 0.39 |
| IRAIN | 0.97(0.68-1.54) | 1.13(0.90-1.73) | 0.12 | MALAT1 | 1.15(0.67-1.60) | 1.01(0.72-1.50) | 0.62 |
| PCAT1 | 0.99(0.80-1.21) | 1.13(0.90-1.40) | 0.06 | RGMB-AS1 | 0.90(0.73-1.46) | 0.92(0.71-1.24) | 0.68 |
| CCAT2 | 0.92(0.65-1.38) | 0.94(0.68-1.13) | 0.81 | PANDAR | 0.93(0.76-1.33) | 0.96(0.68-1.40) | 0.53 |
| Linc01207 | 0.95(0.72-1.30) | 0.87(0.69-1.15) | 0.22 | HNF1A-AS1 | 0.97(0.79-1.21) | 0.83(0.66-1.20) | 0.09 |
